# Mycotic aneurysm of the femoral artery resulting from mismanagement of a pathological femur fracture due to chronic osteomyelitis: a case report

**DOI:** 10.1186/1752-1947-7-8

**Published:** 2013-01-09

**Authors:** Erisa Sabakaki Mwaka, Phillip Mulepo

**Affiliations:** 1 Anatomy Department, School of Biomedical Sciences, College of Health Sciences, Makerere University, PO Box 7072, Kampala, Uganda; 2 Orthopaedics Department, School of Medicine, Makerere University, PO Box 7072, Kampala, Uganda

**Keywords:** Mycotic aneurysm, Osteomyelitis, Pathological fracture

## Abstract

**Introduction:**

Mycotic aneurysms are rarely listed among the possible complications of osteomyelitis of the long bones. To the best of our knowledge this is the first case of chronic osteomyelitis associated with a pathological fracture of the femur and a mycotic aneurysm of the femoral artery.

**Case presentation:**

We present the case of a 13-year-old Ugandan boy who was referred to our hospital with chronic osteomyelitis associated with a pathological fracture of the right femur and a mycotic aneurysm of the femoral artery. He underwent a successful above-knee amputation and is currently undergoing rehabilitation.

**Conclusions:**

Aneurysms associated with chronic osteomyelitis of the long bones are very rare. However, in Africa, where people often still believe in crude traditional remedies, they should be considered among the possible diagnoses especially where acute injuries of the limbs are massaged and manipulated.

## Introduction

Common complications of acute osteomyelitis include chronic osteomyelitis, sterile effusion into adjacent joints, septic arthritis, dislocation of joints, pathological fractures, limitation of joint mobility, bone deformities, septicemia and thrombophlebitis; death can also occur [1].

Aneurysms can occur in almost any artery in the body, but the most common locations are in the abdominal aorta, thoracic aorta, cerebral vessels, and iliac, popliteal, and femoral arteries in descending order of frequency [2]. Isolated true femoral artery aneurysms are relatively rare, and are most commonly found in older men with a strong history of smoking [2]. Infected femoral aneurysms constitute a challenging problem with regard to treatment options, as they are associated with high mortality and morbidity, including limb loss [3]. A high index of suspicion should be present whenever an inflammatory mass appears near a large artery so that prompt treatment is initiated. With the widespread use of antibiotics, the incidence of infected aneurysms has decreased, and arterial trauma has become the most frequent cause [4,5]. In this report we present the case of a patient with a mycotic aneurysm complicating a mismanaged pathological fracture.

## Case presentation

A 13-year-old Ugandan boy of Bantu ethnicity was referred to our hospital for vascular and orthopedic surgical attention. He had a history of bleeding from the right thigh and an inability to use his right lower limb for three weeks.

He had been well until three months prior to referral when he developed a high-grade fever associated with severe pain and progressive swelling of his right thigh for about 11 days, but with no history of trauma. He was taken to a local hospital where a considerable amount of pus was drained from his right thigh. A diagnosis of chronic osteomyelitis of the right femur was made, since it was noticed at surgery that the periosteum had been stripped off the bone. He was put on a six-week course of cloxacillin, the pain reduced significantly and the fever eventually disappeared; however, pus discharge from a sinus on the antero-medial aspect of his right thigh persisted. Three weeks prior to referral to our hospital he slipped on a wet floor, fell, and fractured his right femur. He was taken to a traditional bone setter who started manipulating and massaging his right thigh. After four days of traditional treatment he started to bleed profusely and lost consciousness in the process. He was then rushed to the regional referral hospital where he was transfused with two units of blood, the right lower limb was splinted with a long leg plaster of Paris (POP) back slab and compression dressings applied to the thigh. A radiograph taken at the time showed a pathological fracture with a long sequestrum of the right distal half of the femur and minimal involucrum formation. However, no attempt was made to ascertain the source of the bleeding. He spent three weeks in this hospital, where the staff repeatedly transfused him and applied compressive dressings because he kept on bleeding (though not profusely), in the hope that the bleeding would stop. However this never happened, and our patient had to be referred to our national referral hospital. He had no family history of sickle cell disease or any bleeding disorders, and was human immunodeficiency virus (HIV) negative.

On physical examination, he was toxic, wasted and had a foul smell around him. He had severe pallor of the mucous membranes, was febrile (39°C) and had pedal pitting edema. The right lower limb was splinted in a POP back slab with a bulky blood-soaked compressive padding of the mid-thigh. The right foot was swollen and pale, capillary refill was more than two seconds, the dorsalis pedis and posterior tibial pulses were palpable but very weak.

Full hemogram results were: hemoglobin 5.2g/dL, neutrophils 89 percent, platelets 187×10^3^ cells/mL, erythrocyte sedimentation rate 115mm/hour. Blood culture results were positive for *Staphylococcus aureus* sensitive to ceftriaxone. Radiography results showed a diaphyseal fracture of the femur.

He was immediately resuscitated and prepared for emergency surgery. In surgery, the POP slab and multiple layers of foul-smelling gauze and cotton were removed. Upon removal of the compressive dressing he started bleeding profusely, therefore pressure was applied to the bleeding area and a pneumatic tourniquet applied. There were numerous maggots, a huge hematoma and necrotic tissue on the antero-medial aspect of the distal right thigh. All necrotic tissue was removed and the wound extended for better exploration. A fracture of the distal femoral shaft was found, with the periosteum having been stripped from more than two-thirds of the bone. The femoral artery was friable, and had a ruptured 4cm dilatation (aneurysm) at the level of the fracture.

The femoral artery was ligated and an above-knee amputation of the right lower limb performed. The amputation stump was left open; delayed primary closure was performed three days later. Post-operatively he was transfused and given intravenous ceftriaxone and gentamycin. He did well post-operatively and his wounds healed well, and he was discharged on crutches after 10 days of hospitalization. He was very grateful for our intervention because we alleviated his pain and saved his life. He is currently undergoing rehabilitation in preparation for fitting with a prosthesis. The aneurysmal tissue was sent for histological examination, with the resulting report stating ‘wall of artery with areas of hyaline degeneration and congested vessels’.

## Discussion

Osler introduced the term mycotic aneurysm in 1885 [6]. At that time, cases were usually related to bacterial endocarditis. With the widespread use of antibiotics, the incidence of infected aneurysms has decreased, and arterial trauma has become the most frequent cause [4,5].

The pathogenesis of mycotic aneurysms may include several different mechanisms: septic embolization lodged in the vasa vasorum or vessel lumen; contiguous septic processes extending to the peri-arterial lymphatic vessels and the vasa vasorum of nearby arteries; direct bacterial inoculation at the time of arterial trauma; bacterial infection of an intimal injury, or an atherosclerotic plaque during bacteremia; and self-induced vascular manipulation or iatrogenic causes [4]. In one special circumstance, that of *Salmonella* bacteremia, it is clear that normal arterial intima, which is usually highly resistant to bacterial encroachment, may be invaded by the infectious agent. Frequently, several mechanisms operate simultaneously [4]. In our patient’s case the exact cause of the aneurysm could not be ascertained; however, we postulate that the already vulnerable femoral arterial wall might have been damaged by the bone fragments at the fracture site during the course of manipulation and massage by the traditional bone setter, leading to acute aneurismal dilatation and eventual rupture of the femoral artery.

The diagnosis is confirmed by imaging studies (ultrasound, computed tomography, angiography or magnetic resonance angiography) and, sometimes, mycotic aneurysms are only demonstrable in this way. Mycotic aneurysms are often associated with radiographic findings: the presence of air within the aneurysm, local inflammation, contained rupture or saccular or lobulated aneurysm. We could not carry out all the necessary investigations primarily because our patient was very ill and needed urgent life-saving interventions.

*S. aureus* is the most commonly isolated organism in mycotic aneurysms, with *Salmonella* species, β-hemolytic *Streptococcus*, *Mycobacterium tuberculosis*, *Escherichia coli*, and anaerobic species also identified [1,7,8].

Mycotic aneurysms are associated with high morbidity and mortality rates, and combined therapy results in better outcome [5]. The management of mycotic aneurysms is well established. Extra-anatomic revascularization is recommended, followed by a large excision of the aneurysm and, through a completely separate incision, debridement [9]. Vascular reconstruction by *in situ* implants is possible if the bacterial agent is not multi-resistant, if the infectious process is not very extensive, and if complete debridement is feasible. In such cases, venous grafts, arterial autografts, or arterial homografts are used [7,10]. The risk of re-infection seems to be reduced by covering the prosthesis and all sutures with a pediculated omental or muscular flap. If these techniques of revascularization fail, ligation of the arterial axes may become necessary. Ligation carries with it a 25 percent risk of ischemia and amputation [11]. Though indicated, we could not venture into vascular reconstruction as a limb-salvaging procedure because of a lack of the necessary expertise.

Bone infection in primary osteomyelitis is usually hematogenous, however as a consequence of the HIV/acquired immunodeficiency syndrome (AIDS) pandemic osteomyelitis has been reported as a complication of pyomyositis [12]. To avoid serious complications it is imperative to recognize, diagnose and treat osteomyelitis effectively with appropriate antibiotics. Unfortunately the diagnosis is often difficult during the first 10 to 14 days when obvious symptoms such as fever, pain, local swelling, malaise, loss of appetite, vomiting and local heat may be unremarkable. At this stage radiographic changes may remain negative. The results of blood cultures for diagnosis may be uninformative, but should be repeated as often as possible, especially during peaks of pyrexia. Diagnosis of osteomyelitis in Uganda is usually performed late because most clinicians always first think of common causes of fever such as malaria, enteric fever and upper airway infections. Some cases are diagnosed late because they first seek the services of traditional healers.

Aneurysms are associated with several complications; according to Levi *et al*. distal embolization occurs in up to 26 percent of cases, and acute thrombosis occurs in around 15 percent of cases. Rupture is uncommon and incidence varies between 10 percent and 14 percent of cases [2]. Another review by Fluckiger *et al*. shows the risk of complications such as rupture, thrombosis and embolization to be above 50 percent. In their report, acute dilatation and rupture occurred in 34.5 percent of cases [13].

Mycotic aneurysms carry a poor prognosis, so it is mandatory to obtain adequate material for culture and sensitivity testing to enable a suitable combination of antibiotics to be selected. Common general principles of management include control of hemorrhage if present, the obtaining of microbial cultures, wide debridement of all infected tissue, consideration of arterial reconstruction (usually autogenous tissue, preferably through uninfected tissue planes), and continued antibiotic therapy throughout the post-operative period.

## Conclusions

Aneurysms associated with chronic osteomyelitis are very rare. However, in Africa where people still believe in crude traditional remedies, they should be considered among the possible diagnoses especially where acute injuries of the limbs are massaged and manipulated. Chronic osteomyelitis is not uncommon in the developing world, therefore whenever faced with pathological fractures secondary to an infective process vascular injuries and aneurysms should always be borne in mind.

### Consent

Written informed consent from the patient’s next-of-kin and the patient were obtained for publication of this case report and any accompanying images. A copy of the written consent is available for review by the Editor-in-Chief of this journal.

## Competing interests

The authors declare that they have no competing interests.

## Authors’ contributions

MES and MP performed the emergency surgery on our patient. MP complied all the medical case notes and investigations. MES wrote the final manuscript. All authors read and approved the final manuscript.
